# Hydrogel Innovations in Biosensing: A New Frontier for Pancreatitis Diagnostics

**DOI:** 10.3390/bioengineering12030254

**Published:** 2025-03-03

**Authors:** Prerna Sutar, Atharv Pethe, Piyush Kumar, Divya Tripathi, Dipak Maity

**Affiliations:** 1 School of Health Sciences and Technology, UPES, Bidholi Campus, Dehradun 248007, Uttarakhand, India; 2 Integrated Nanosystems Development Institute, Indiana University Indianapolis, Indianapolis, IN 46202, USA; 3 Department of Chemistry and Chemical Biology, Indiana University Indianapolis, Indianapolis, IN 46202, USA

**Keywords:** hydrogel biosensor, pancreatitis, biomarker sensing, wearable biosensors, diagnostic technologies, biocompatible sensors, point-of-care diagnostics

## Abstract

Pancreatitis is a prominent and severe type of inflammatory disorder that has grabbed a lot of scientific and clinical interest to prevent its onset. It should be detected early to avoid the development of serious complications, which occur due to long-term damage to the pancreas. The accurate measurement of biomarkers that are released from the pancreas during inflammation is essential for the detection and early treatment of patients with severe acute and chronic pancreatitis, but this is sub-optimally performed in clinically relevant practices, mainly due to the complexity of the procedure and the cost of the treatment. Clinically available tests for the early detection of pancreatitis are often time-consuming. The early detection of pancreatitis also relates to disorders of the exocrine pancreas, such as cystic fibrosis in the hereditary form and cystic fibrosis-like syndrome in the acquired form of pancreatitis, which are genetic disorders with symptoms that can be correlated with the overexpression of specific markers such as creatinine in biological fluids like urine. In this review, we studied how to develop a minimally invasive system using hydrogel-based biosensors, which are highly absorbent and biocompatible polymers that can respond to specific stimuli such as enzymes, pH, temperature, or the presence of biomarkers. These biosensors are helpful for real-time health monitoring and medical diagnostics since they translate biological reactions into quantifiable data. This paper also sheds light on the possible use of Ayurvedic formulations along with hydrogels as a treatment strategy. These analytical devices can be used to enhance the early detection of severe pancreatitis in real time.

## 1. Introduction

Acute pancreatitis (AP) is an inflammatory condition of the exocrine pancreas associated with tissue damage and necrosis. The severity of the disease ranges from moderate, self-limiting cases to severe forms that lead to extra-pancreatic organ failure and can be fatal [1]. Currently, no pharmacological drugs are available to alter the progression of the disease; the only viable option is early disease management within 72 h [2]. The most frequent cause of acute pancreatitis is gallstones, which obstruct the pancreatic duct, exposing the pancreas to biliary substances [3]. Alcohol misuse is the second most common cause, typically involving heavy drinking (approximately 4–5 drinks per day for over 5 years) [4]. Alcohol consumption and cigarette smoking often coexist, and recent research indicates that these behaviors significantly impact health [5]. Beyond alcohol misuse, smoking is an independent risk factor for acute, recurrent, and chronic pancreatitis [6]. Additionally, even mild or moderate hypertriglyceridemia (HTG) is known to aggravate acute pancreatitis and is thus recognized as a risk factor [7]. Acute pancreatitis occurs in an estimated 15% to 20% of individuals with severe hypertriglyceridemia (triglyceride levels > 1000 mg/dL) [8].

Medication-associated pancreatitis is less common, accounting for fewer than 5% of cases, but has been linked to several drugs. For instance, a 36-year-old man developed severe acute pancreatitis four days after consuming orlistat, a lipase inhibitor for obesity [9]. Other drugs strongly associated with acute pancreatitis include azathioprine, 6-mercaptopurine, didanosine, valproic acid, ACE inhibitors, and mesalamine [10]. In pediatric cases, pancreatitis is much less common but has been linked to genetic influences. Mutations affecting digestive enzymes are key contributors to the condition [11].

The observable symptoms in a person suffering from acute pancreatitis are abdominal pain with a burning sensation that radiates to the back muscles along with elevated serum levels of amylase and lipase up to three times the normal concentrations [12]. A general protocol for detection includes medical imaging techniques. Ultrasound imaging or MRI is conducted first to rule out possibility of gallstones and pancreatic lesions, followed by a CT scan if earlier results are inconclusive, whereas there is no need for imaging techniques if serum concentrations of lipase have increased by a factor of three [13]. Since early detection can save the lives of people suffering from pancreatitis, the therapeutic need to develop a point-of-care testing device means all the difference in saving lives. Gold-standard analytical methods for the detection of serum amylase and serum lipase levels are based on spectrophotometry, turbidimetry, high-performance liquid chromatography, and ELISA-based assays but are not very helpful due to their operational times and reliance on skilled expertise [14]. There have been numerous reports of nanomaterial-based biosensors developed for the detection of several biomarkers of AP, such as amylase, trypsin, serum lipase, triglycerides, and serum creatinine, from serum and urine samples, but their deployment in vivo has not been fully functionalized due to their possible toxicity, thereby creating a demand for biocompatible nanomaterials [15]. Hydrogels are 3D crosslinked polymers and excellent bio mimics of hydrated biological tissues, and their ability to react to changes in temperature, redox potential, pH, light, and ionic changes makes them quite useful for biosensing applications [16,17]. There have been reports on the development of urine-based biosensors based on hydrogels for the detection of biomarkers by the use of diapers, even achieving LODs for creatine and uric acid as low as 0.59 µM and 69 µM, respectively [18]. Thereby there may be new avenues that can be explored for innovate sensing strategies based on the fact that hydrogel-based biosensors can be applied for AP detection by analyzing samples invasively as well as non-invasively. Evidence for this is the development of hydrogel-based microneedles, which can be used for sampling as well as controlled drug delivery, making them suitable for the minimally invasive sampling of tissues [19]. The diagnosis of pancreatitis is difficult due to the invasiveness of biopsy procedures, which risk damage to the organ, which can lead to increased severity of the disease. Therefore, the development of a biosensor that can detect potent biomarkers of pancreatitis can bring about a revolution in the early detection of disease and thereby have potential lifesaving applications. A strategy based on invasive sampling and ex vivo detection for confirmation can also be developed using differential biorecognition elements and transducing technologies for biosensing.

## 2. Pancreatitis

### 2.1. Causes of Pancreatitis

**Figure 1:**
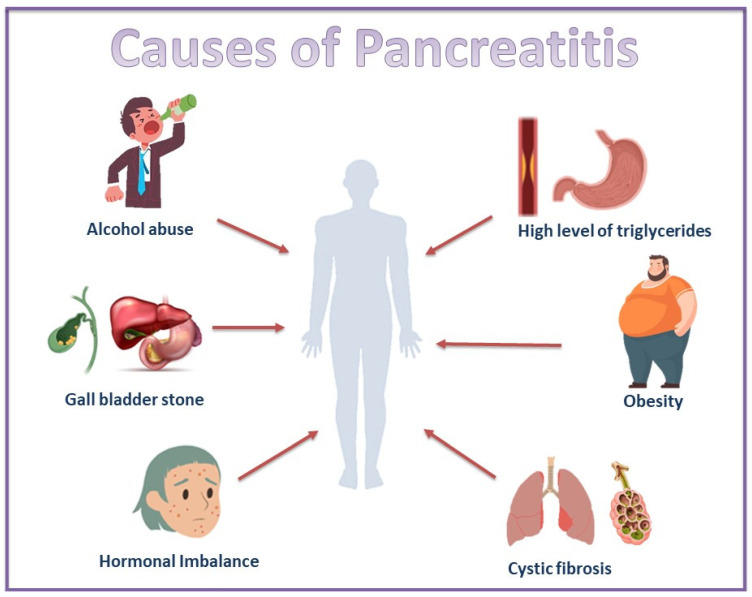
Various causes of acute pancreatitis. These are mostly associated with lifestyle choices, and a known disease co-relationship exists between the habit of drinking and obesity, which are known to affect the health of individuals.

Acute pancreatitis is the inflammation of the exocrine pancreas, which leads to the necrosis of pancreatic tissue. If not resolved spontaneously within days, it may result in extra-pancreatic organ failure, which can lead to fatal consequences [20]. Due to the lack of therapeutic agents currently in use for the treatment of this condition, fluid resuscitation and supportive care to prevent the disease from becoming severe have been adopted. Numerous factors contribute to the condition, such as autoimmune pancreatitis, hypertriglyceridemia, and lifestyle factors, as shown in Figure 1; post-endoscopic retrograde cholangiopancreatography (ERCP), medications, genetic risk (mutations in the CFTR (cystic fibrosis trans-membrane conductance regulator protein) and SPINK1 (serine protease inhibitor Kazal type 1) genes that cause gain of function), and pancreatic duct injury are also among the causes [21].

Hyperglyceridemic pancreatitis is the third leading cause of acute pancreatitis, and therefore, conditions that could lead up to this should be avoided at all costs [22]. There are a lot of other metabolic factors like hyperglycemia, dyslipidemia, hypertension, and obesity that, when observed in an individual’s lifestyle, increase their risk of pancreatitis [23]. The role of obesity in cases of increased body mass index has posed a negative impact and is a possible prognostic factor for acute pancreatitis, as increased levels of pro-inflammatory cytokines and adipokines are related to an increase in intra-pancreatic fat [24]. Conditions like fatty liver, diabetes mellitus, and hypertension are some factors that contribute to the elevation of the risk of severe acute pancreatitis (AP) [25]. Mechanical causes, toxins, and metabolites that are potential causes of pancreatitis are included in Table 1.

### 2.2. Etiology and Pathogenesis of Pancreatitis

The most common theory about the pathophysiology of pancreatitis holds that a disruption in the regulation of pancreatic digestion enzymes by pancreatic acinar cells causes inflammation that is either initiated or maintained [26,27]. The digestive enzymes synthesized in pancreatic acinar cells are normally maintained in an inactive form (i.e., zymogens such as trypsinogen), and while in the acinar cells and in the draining duct, they are converted to an active form (such as trypsin) upon entry to the gut lumen by enterokinase or trypsin itself [28]. This information is crucial to understand the mechanism of disruption. Premature intra-acinar cell activation of the dormant enzymes can be attributed to many factors that may lead to the auto-digestion of acinar cells, which can then trigger subsequent events that result in persistent pancreatic inflammation [29]. Trypsinogen autoactivation, the activation of trypsinogen by lysosomal hydrolase cathepsin B (CatB) caused by the shifting of trypsinogen into cellular compartments rich in proteases (lysosomes), and imbalances between degrading and activating acinar cell cathepsins are some of the mechanisms of such premature and inappropriate activation of normally inactive zymogens that have been proposed [30]. It should be mentioned that elevated intracellular calcium concentrations are necessary for the activation of pancreatic proteases; as a result, abnormalities in these concentrations may be a contributing factor to pancreatitis [31].

The breakdown and recycling of cytoplasmic organelles and long-lived proteins occurs during autophagy [32]. Targeted proteins are engulfed in double-membrane autophagic vacuoles during this process, and when the autophagic vacuoles fuse with lysosomes, they become exposed to proteolytic breakdown. The strongest evidence for this theory comes from studies conducted on mice that have specific deletions of important autophagy genes. For example, Ref. [33] demonstrated that mice with a pancreas-specific autophagy-related 5 (ATG5) deficiency develop chronic pancreatitis and exhibit endoplasmic reticulum stress in their acinar cells.

Since the ablation of scaffold protein p62 partially occurs, corrected alterations due to the deletion of a protein kinase inhibitory-kB kinase alpha (IKKα), it was assumed that these pathogenic changes, along with the presence of elevated oxidative and endoplasmic reticulum stress, were caused by the accumulation of protein aggregates dependent on p62, a protein ordinarily removed by autophagy [34]. The exact mechanism by which the IKKα deletion affects autophagy remains relatively unknown; however, since IKKα interacts with ATG16L2, a malfunctioning autophagy protein, this could be the root cause of the autophagy deficiency.

Pancreatic acinar cell death, a prominent characteristic of pancreatitis, may result from necroptosis or apoptosis [35]. Since necrosis is the predominant cell death mechanism in related mouse models of cerulein-induced pancreatitis, and apoptosis is the main process in rat models, there may be some species-dependent variation in which of these death processes is at play [36]. The fact that apoptosis and necrosis have different but similar molecular mechanisms is probably connected to these surprising species differences. The tumor necrosis factor-α-mediated activation of TNF receptor type 1-associated death domain protein (TRADD)/Fas-associated protein with death domain (FADD) or receptor-interacting protein kinase 1 (RIP1)/RIP3 is the most common mechanism by which apoptosis is mediated under pathological conditions. This is followed by the activation of caspase-8, commonly known as the “executioner” molecule [37]. Necroptosis can be triggered by a variety of stimuli that induce TNF-α or, conversely, TLR3 or TLR4 signaling; however, it is unclear under what conditions this type of cell death takes place instead of apoptosis. Since necroptosis is inhibited by caspase-8, it is plausible that necroptosis happens when caspase-8 is either not created or is inactivated; on the other hand, since RIP3 is specifically implicated in necroptosis, it is also feasible that necroptosis occurs when RIP3 is strongly activated. According to these theories, in rat cerulein pancreatitis, caspases are triggered, and RIP is cleaved; however, in this model, an endogenous caspase inhibitor (XIAP) is degraded [38].

However, research on inflammation strongly suggests that it most likely stems from the release of damage-associated molecular patterns (DAMPs) from pancreatic acinar cells that are dying. These DAMPs then trigger inflammation when the innate immune system’s pattern recognition receptors (PRRs) are activated. Several types of DAMPs have been identified as pancreatitis-associated “danger signals” (i.e., DAMPs) in human and experimental pancreatitis. These types of DAMPs include high-mobility group box protein 1 (HMGB1), self-DNA, nucleosomes (DNA coiled around histone octamers), and adenosine triphosphate [39]. Out of all of them, HMGB1 is particularly noteworthy because [40] found that animals and individuals suffering from acute pancreatitis had much higher levels of this DAMP.

Hoque et al. [41] clarified a potential molecular mechanism for cerulein-induced pancreatitis that is mediated by a DAMP–PRR interaction. These authors demonstrated that pancreatic macrophage TLR9, an endosome-linked PRR sensitive to bacterial CpG DNA and self-DNA, is activated by self-DNA produced from dying acinar cells during the early stages of cerulein-induced pancreatitis. Simultaneously with this, released adenosine triphosphate activates the purinergic receptor P2X7, which subsequently combines with TLR9 activation to activate the NLRP3 inflammasome and convert pro-IL-1β to mature IL-1β. The AIM2 inflammasome, which is lacking in melanoma 2, has been linked to a DAMP–PRR interaction in pancreatitis, according to recent reports. Nucleosomes, which were previously indicated as potential pro-inflammatory DAMPs in pancreatitis, were the stimulating DAMPs in this instance. According to [42] pertinent investigations, nucleosome activation of the AIM2 inflammasome and IL-1β production was necessitated by the phosphorylation of dsRNA-activated protein kinase mediated by the receptor for advance glycation end products, or RAGE. Furthermore, the authors discovered that under these conditions, L-arginine-induced pancreatitis is less common in AIM2- and RAGE-deficient animals, and HMGB1 expression an inflammasome products is also less common [43].

**Figure 2:**
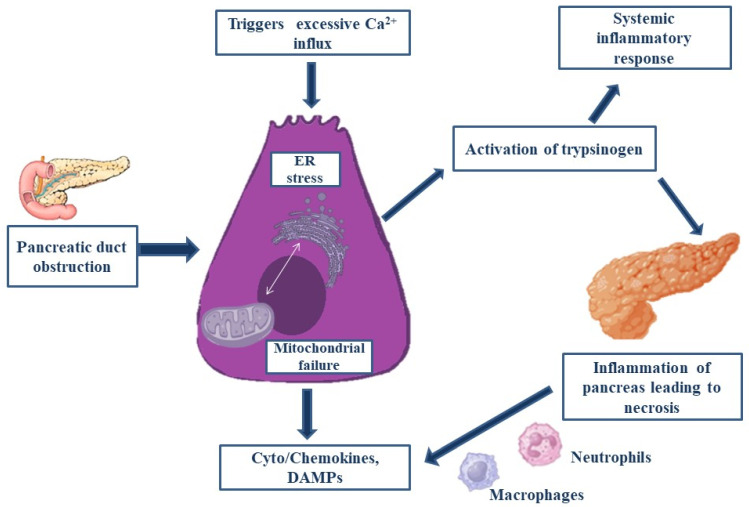
Summary of the causative agents responsible for increasing the risk of pancreatitis.

When significant local and systemic problems are evident, acute pancreatitis (AP), a type of severe pancreatitis, has a high morbidity and death rate. Numerous studies carried out in the past few decades have shown that identifying individuals who are at risk of complications or death depends heavily on what happens in the first 24 h following the onset of symptoms. Gallstones and alcohol are the two most common causes, yet the exact reason is unknown due to its complicated etiology [44]. None of the mechanisms that have been proposed to explain the pathophysiological process of acute pancreatitis are particularly clear. A few of the theories include the early activation of trypsin by the acinar and ductal membrane, the attraction and activation of leukocytes, and the recruitment of adhesion molecules, cytokines, and oxygen free radicals, all of which result in microcirculatory damage and mitochondrial dysfunction, which is depicted in Figure 2 [45]. The characteristic that all mechanisms have in common is an overreaction to inflammation.

Due to causes like the pancreatic duct obstruction and excessive calcium ion influx, cellular abnormalities like endoplasmic reticulum (ER) stress, mitochondrial failure, and autophagy dysfunction are seen, in response to which chemokines and DAMPs are released. These abnormalities are also responsible for the activation of trypsinogen, which produces inflammation in the pancreas, leading to necrosis, and ultimately, a systemic inflammatory response is observed.

**Figure 3:**
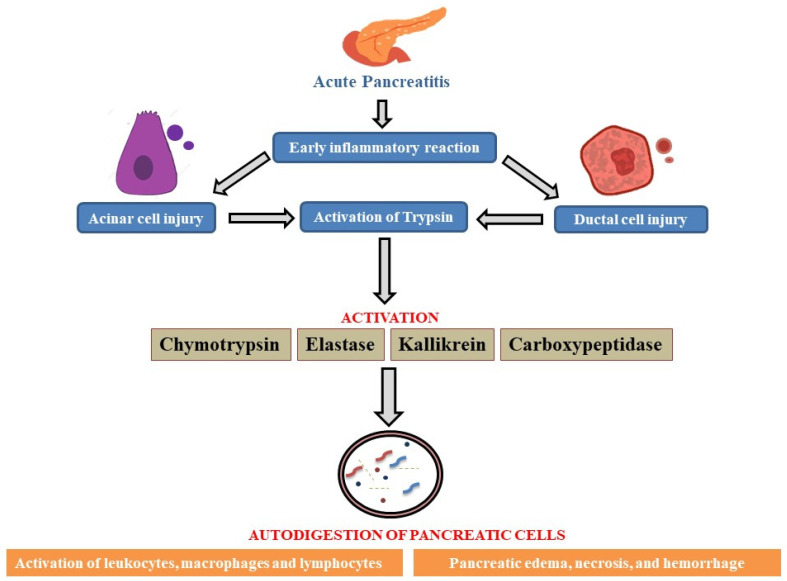
A schematic overview of acute pancreatitis’ pathogenesis. The onset of AP via early inflammatory response leads to pancreatic cellular injury followed by the downstream activation of enzymes, which promotes the auto-digestion of pancreatic cells, worsening the patient’s condition with symptoms such as pancreatic edema, tissue necrosis, and hemorrhage caused by rapid inflammation due to activation of leukocytes, macrophages, and lymphocytes.

As there is relative difficulty in obtaining pancreatic tissue, scientists have shifted their attention to studying the disease pathology via animal models to investigate the molecular elements of acute pancreatitis pathogenesis [45]. The disparate outcomes from various animals and models subjected to comparable etiologies further compound the problem. The most widely accepted idea about the primary mechanism underlying the start of pancreatic tissue auto-digestion and the subsequent onset of systemic and local inflammatory processes is the early activation of trypsin. Its progression is divided into three phases: local inflammation, generalized inflammatory response, and multiorgan dysfunction [46]. The pathophysiology of AP has been the subject of numerous publications; the exact mechanism behind this illness is still unknown, but a theoretical pathology has been established, as illustrated in Figure 3 [47].

### 2.3. Biomarkers of Pancreatitis

An essential inflammatory cytokine involved in the pathophysiology of acute pancreatitis is tumor necrosis factor (TNF)-α, along with IL-1β, IL-6, IL-8, and IL-10 as the most relevant biomarkers [48], which damages acinar cells directly and causes necrosis, inflammation, and edema. This cytokine is the primary immune response mediator and is believed to be the first cytokine released. The onset of experimental acute pancreatitis causes an increase in TNF-α expression in the pancreas. It is commonly recognized that interleukin plays a crucial role in the early stages of the pancreatic acute inflammatory process [49]. In a study to assess the severity of acute pancreatitis, it was discovered that the interleukin-1 receptor antagonist had the best accuracy among many indicators and that interleukin-1 levels suggested severe acute pancreatitis on admission with accuracy similar to that of interleukin-6 (82% and 88%, respectively). An integrated electrochemical magnetic immunosensor based on gold nanoparticles and graphene was developed, which had an LOD of 0.4 pg/mL for human serum samples [50]. Wang et al. [51] confirmed that after administering L-arginine, there was a significant increase in the concentration of TNF-α in the pancreas. The overproduction of reactive oxygen species (ROS) could be the cause of this, as it triggers the activation of nuclear factor kappa B (NF-κB), leading to the overexpression of multiple inflammatory cytokines, especially interleukin (IL)-1β and TNF-α. Patients with severe acute pancreatitis have been discovered to have elevated levels of TNF-α receptors, which are markers of TNF-α activity [52]. It has also been demonstrated that TNF-α inhibition lowers mortality and lessens the severity of experimental acute pancreatitis. An electrochemical sensor was also developed for the simultaneous detection of TNF-α and IL-1β with carbon nanotubes functionalized with 4-carobyphenyl in conjugation with a sandwich-type immunoassay for each cytokine, with an LOD up to 0.85 pg/mL and 0.38 pg/mL, respectively [53].

The primary trigger for acute-phase protein synthesis in the liver is interleukin-6, which also serves as the primary mediator in the production of hepcidin, fibrinogen, and C-reactive protein (CRP) [54]. Numerous studies have also confirmed the importance of IL-6 in the early and accurate prediction of pancreatitis severity. CRP is a positive acute-phase reactant that the liver produces in response to inflammation and infection [55]. It is triggered by cytokines such as interleukin-6, and its blood level rises considerably with time. Particularly in cases involving inflammation, it can be utilized for diagnosis, prognosis, therapy monitoring, and death prediction. A reusable and optimized label-free biosensor for the detection of CRP was developed with a limit of detection of 176 pM from whole blood or serum samples using electrochemical impedance spectroscopy [56]. A cost-effective, label-free point-of-care device was developed based on electrochemical methods for the detection of CRP from enriched plasma [57].

Pro-peptide pro-calcitonin (PCT) is produced by thyroid gland G-cells and hepatocytes [58]. Several investigations have confirmed its function as an early biochemical marker in infection, sepsis, and multiorgan failure [59]. Sepsis, infected pancreatic necrosis, and multiorgan failure are known to be linked to severe acute pneumonia, and preclinical CT (PCT) can be utilized to assess acute pancreatitis early on [58]. Among the serine proteases present in neutrophil granules, polymorphonuclear (PMN) elastase, proteolytic enzymes, cationic peptides, reactive oxygen species, and eicosanoids are just a few of the microbicidal compounds that are released when granulocytes infiltrate and become activated as a first-line defense after tissue damage [60]. The early stages of acute pancreatitis also involve this process [61]. Endothelial activity is marked by soluble E-selectin (sES), while endothelial damage is marked by soluble thrombomodulin (sTM). The endothelium is harmed by the production of elastases by activated neutrophils during acute pancreatitis [62]. A novel approach for the estimation of thrombomodulin based on TM protein-modified gold nanoparticles and an antibody bound to glucose oxidase and gold nanoparticles was able to determine TM concentrations with an LOD of 5.7 ng/mL [63].

Hemoconcentration could be a sign of pancreatic microcirculation insufficiency, leading to necrosis. It has been suggested that hemoconcentration at admission, as determined by initial hematocrit, is a helpful prognostic indicator for acute pancreatitis [64]. It has been demonstrated that there is a higher chance of necrosis and severe pancreatitis in cases of early hemoconcentration [65]. It has been confirmed that increased renal permeability occurs following ischemia, burn injuries, trauma, and surgery, which causes minimal proteinuria [66]. In many diseases, the degree of proteinuria is correlated with severity and prognosis. A urine dipstick can identify this signal, providing quick and affordable findings. Several investigations have confirmed its function as an early biochemical marker in infection, sepsis, and multiorgan failure [67].

PCT can be utilized as an early diagnostic test for acute pancreatitis (AP) because severe acute pancreatitis is known to be associated with sepsis, infected pancreatic necrosis, and multiorgan failure. It has been discovered that endothelin I (ET-1) levels are elevated in acute pancreatitis patients, and these levels strongly correlate with the severity of the condition [68]. The blood concentration of albumin, a negative acute-phase protein produced by the liver, falls when inflammation increases [69]. Because of the connection between inflammation and malnutrition, albumin has also been investigated in relation to the degree of inflammation, the prognosis of the disease, and death. It has also been shown that low ionized calcium (Ca^2+^) levels in blood serum are a significant factor for the identification of patients with severe pancreatitis. In addition to these biomarkers, other biological factors such as intercellular adhesion molecule (ICAM) [70], histone protein [71], angiopoietin-2 [72], serum Cys-C [73], creatinine [74], and hemoglobin, to mention a few, are also likely to be strong indicators of pancreatitis [75].

A multiplexed enhanced fluorescence microarray immunoassay with a gold nanostructured island substrate that enhanced near infrared florescence signals for the detection of ICAM achieved 202.2-fold fluorescence signal enhancement over traditional methods [76]. An SPR biosensor was developed for the identification and quantification of angiopoietin-2, with an LOD up to 0.01 pg/mL [77]. Similarly, a POC device was developed for the determination of urinary creatinine via pH-responsive and polymer-modification-based biosensing, coupled to a smartphone [78].

Summarizing potential biomarkers for the detection of pancreatitis, Table 2 mentions cut-off amounts and the time of testing for these biomarkers. By incorporating these biological components with their appropriate substrates, we can modify a biosensor technology to aid in the early identification of pancreatitis.

## 3. Hydrogel-Based Biosensors

Hydrogels are three-dimensional, water-swellable structures that are created through physical crosslinking (non-covalent interactions) or chemical crosslinking (covalent bonds [87]. Their molecular interaction with biological components, their regulating viscoelastic properties, their reactivity to external stimuli, their antifouling characteristics, and the availability of numerous well-known synthesis methods for integrating bio-receptors into their highly wet structure are just a few of the features that have made them popular for biosensing applications.

**Figure 4:**
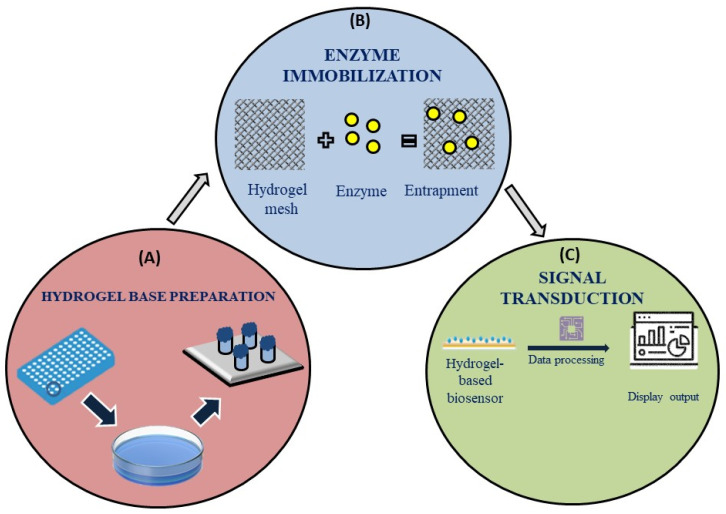
A graphical representation of a basic working model of a hydrogel-based biosensor. The hydrogel-based sensors operate via (**A**) the creation of a hydrogel base, (**B**) the immobilization of enzymes and their reaction with target biomarkers in the test fluid, and (**C**) the display of signals using a sophisticated signal transduction apparatus.

Hydrogels are generally biocompatible and mimic hydrated biological tissues, which presents an amazing possibility for their use as sensing system [88]. As such, they may have applications in vivo. Moreover, the synthetic flexibility of these gels enables their chemistry to be fine-tuned to provide desired stimulus responses. These responses provide both indirect (alterations in UV absorption characteristics) and direct (visible color changes) read-outs through degradation and swelling/deswelling [89]. Although numerous nanomaterials have been developed for improving the performance of biosensors, they still meet challenges like a lack of selectivity, sustainability issues, high costs and complex processes of fabrication, and, more importantly, toxicity owing to changes in the material type [90]. Therefore, utilizing their excellent properties in contrast to the mentioned problems, biosensors based on hydrogels can be a viable option for enhancing their effectiveness. Figure 4 mentions the basic working model of a hydrogel-based sensor, including hydrogel base preparation, enzyme immobilization, and signal transduction. However, the monomer utilized for hydrogel synthesis as well as the preparation techniques employed in its creation determine the properties and possible uses of hydrogels [91]. Although hydrogels exhibit great variability in their reactions to a wide range of stimuli, their use in sensing applications necessitates the development of a signal transduction system capable of converting the hydrogel response into a signal that can be understood. An example is the color shift observed in hydrogels containing embedded nanoparticles or micro-particles when glucose was present in a biosensor for detecting pancreatitis [92]. As a result of their many uses, electrochemical-based biosensors have emerged as the most widely utilized kind of biosensors.

Hydrogels have shown promise as an immobilization matrix for biosensing components in electrochemical-based biosensors [93]. Their three-dimensional design increases the amount of recognition factors by offering a larger surface area. Furthermore, hydrogels function well in physiological settings, which enables them to monitor biological events in vivo with little interference from the biological activity. Liquid crystal (LC)-based sensors, due to their advantageous properties of simplified operations, low cost, and high sensitivity, have a shorter operation time. The molecules present at the LC interface can sense the biomarker molecules, amplify them, and transduce them into optical signals, which can be detected. LC-based biosensors have been curated to detect different types of biomolecules like DNA, enzymes, and proteins [94]. A peptide hydrogel-based biosensor was developed for the detection of HER2 from human serum samples and showed high specificity, with an LOD of 45 pg/mL [51]. However, it is always necessary to perform elaborate sample preparation and multiple washing steps to acquire proper results in the detection of biomolecules. Therefore, there is an essential demand to develop a biosensor that can minimize such steps and make the detection of pancreatitis easier.

### Hydrogel as a Possible Therapeutic Carrier of Ayurvedic Medicine

An ancient form of healthcare system that incorporates several dimensions of life to control the onset of diseases via herbal formulations accompanied with diet and lifestyle practices is Ayurveda [95]. Numerous formulations exist and are being validated, and Ayurvedic medicines for managing type 2 diabetes mellitus have been demonstrated [96]. The persistent issue with Ayurvedic medicine is the low biocompatibility of the bioactive ingredients present in the herbal formulations along with slow diffusion rates; these problems, however, can be mitigated by the use of nanotechnology through effective targeted and non-targeted drug delivery [97].

A balanced diet and controlled lifestyle, along with an Ayurvedic herbo-mineral formulation (HMF), have been used to treat pancreatitis patients using processed sulfur, copper, and mercury to create the HMF. This formulation could achieve a 93% reduction in the frequency of AP incidents and a 97% reduction in emergency hospitalization [98]. In another report by [99] a 22-year-old nonalcoholic male patient experiencing AP diagnosed by magnetic resonance cholangiopancreatography was administered a formulation based on *Pittaja gulma*, and after a period of 15 days, his serum markers were regularized. As reported by [100], herbal medicines can be incorporated into hydrogels, as they were able to use a hydrogel patch for bone setting.

**Figure 5:**
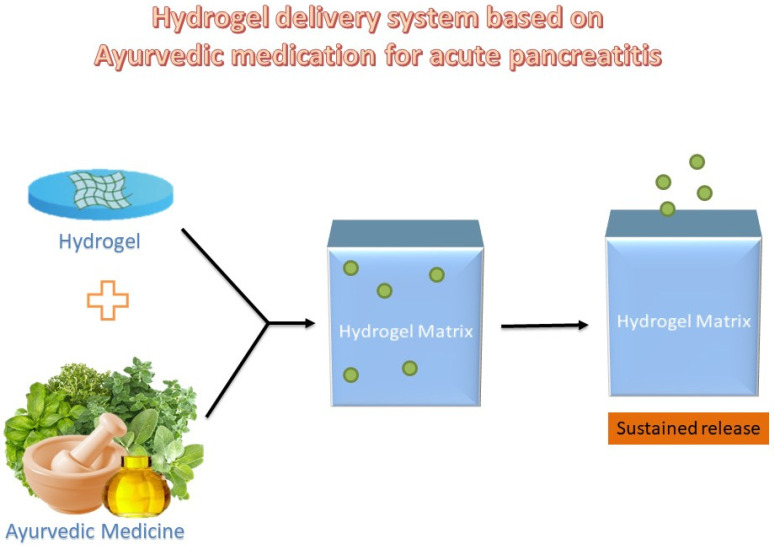
A possible method for the drug delivery of ayurvedic formulations via hydrogels.

To the best of our knowledge, since there have not been any attempts to treat illnesses using Ayurvedic formulations in hydrogel-based carriers (Figure 5), this area can be investigated as a potential alternative to traditional medical treatments. Moreover, Ayurvedic formulations in conjunction with modern medicine can possibly provide better therapeutic strategies via herbal medication. There have not been attempts to use Ayurvedic formulations via hydrogel-based carriers for the treatment of diseases; therefore, this field can be explored to identify alternative remedies for treating diseases.

## 4. Types of Hydrogels

The field of medical diagnostics and research was revolutionized by the use of hydrogels due to their unique properties. They consist of chemically or physically crosslinked polymers, which create porous, hollow spaces with the ability to swell, which allows them to encapsulate various drugs and biomolecules [101]. Biomolecular recognition components are hosted by bio-receptors to uniquely recognize a specific occurrence within a bio-system. Stable bio-receptor immobilization, surface bonding techniques, blocking nonspecific protein adsorption on the hydrogel surface, probe density, flexibility, and swelling kinetics are critical factors in the design of this kind of biosensor [102]. The development of hydrogels relies on sol–gel chemistry which is able to produce a solid-state material from precursors by ensuring that the randomness of molecules in the solution remains captured [103]. Bio-fabrication by using silyated bioinks polymerized by the sol–gel process is a great option to create biocompatible hydrogels [104]. Using inorganic sol–gel polymerization, a cellular-based hydrogel scaffold was created using 3D bioprinting with polymers such as hydroxpropyl methyl cellulose with the claim that this preparation methodology can be applied to any type of multisilylated biopolymer for the formation of various types of bioinks.

### 4.1. Polyvinyl Alcohol

Polyvinyl alcohol (PVA) has shown promising biocompatibility, along with features like high mechanical strength, a low friction coefficient, and suitable water content, plus the ability to be 3D printed, making it ideal for biomedical applications [105]. PVA hydrogels can resemble soft tissue and reduce inflammation and fibrosis, which is essential for implantable sensors because of their flexibility and stability in a variety of environmental circumstances [106].

### 4.2. Polyethylene Glycol

Because of its low interfacial energy, polyethylene glycol (PEG), a hydrophilic biomaterial, offers strong antifouling capabilities that prevent the attachment of proteins and cells to its surface [107]. PEG is frequently utilized as a biosensor with antifouling properties due to its biocompatibility [108]. PEG and its blends have been used in recent research to create mass-based, electrochemical, and optical biosensors [109].

### 4.3. Polyacrylate Families

Sensation-responsive hydrogels, such as polyacrylic acid, poly-hydroxy-ethyl methacrylate, polyacrylamide, and poly(isopropylacrylamide), are primarily employed in biosystems for temperature and pH sensing [110]. Ionic hydrogels are hydrophilic materials that can swell and de-swell in response to changes in their environment because of their dependence on charged group density [111]. Recently, adhesive and conductive hydrogels based on polyacrylate composites were developed and could potentially function as biosensors [112]. Fabrication patterning techniques have recently been recognized as a crucial focus of development for the increased usage of ionic hydrogels’ sensing capabilities, particularly when they are employed without using bio-receptors that have been immobilized.

### 4.4. Biologically Originated Hydrogels

Biomaterials possessing gel-forming qualities include polysaccharides and polypeptides such as hyaluronic acid, agarose, chitin and chitosan, cellulose, dextran, and alginate [113]. These hydrogels with biological origins have special qualities like hydrophilicity, biocompatibility, heavy metal ion chelation, strong protein affinity, and simple surface chemical modification made possible by their easily obtainable materials, inexpensive preparation, suitable mechanical qualities, and reactive functional groups. Their production techniques, even in tiny geometries, makes them appealing for biosensor uses [114]. The tissue-regenerative properties of biologically originated hydrogels have also been well documented [115].

### 4.5. Electro-Conductive Hydrogels

Because of their special qualities, electro-conductive hydrogels comprising hydrated structures with electrical functionality have drawn interest in the field of biomaterials [116]. Such hydrogels have porous structures that offer a wide surface area with higher diffusivity and conductive polymers that help transport electrons across the interface. These hydrogels are important in the field of biosensor technology because of their capacity to conduct electrons with flexibility and processability, as well as the capabilities they acquire through chemical changes [58]. When creating conductive hydrogels, the most popular conducting polymers utilized are polypyrrole, polyaniline, and poly-(ethylene-dioxy thiophene). Due to their redox activity, electrochemical enzyme-immobilized biosensors react to the bio-system environment in a way that causes electrons to transfer across the electro-conductive hydrogel, producing a current or changing the potential to generate voltage [117]. Their high-sensitivity and -specificity monitoring of small molecules, like cholesterol and glucose, presents significant prospects for early therapy, primary diagnosis, and improved chronic illness management. A few specifications of hydrogel-based biosensors for the detection of tiny molecules are shown in Table 3.

## 5. Applications of Hydrogel-Based Diagnostic Tools and Hypothetical Application Strategies for Pancreatic Diagnosis

Advancements in biomedical devices and large hydrogel-based sensors offer a promising solution for point-of-care examinations, providing rapid, reliable, and user-friendly diagnostic tools directly at the bedside or in remote settings. Hydrogel-based biosensors leverage the interaction between the target biomarker and a functional hydrogel matrix to produce a measurable signal. By keeping a moist environment, alginate, a polysaccharide with a high absorption capacity, helps to reduce wound fluids and enhance wound healing. By utilizing ionic crosslinking, researchers were able to effectively create an alginate–hyaluronan hydrogel, which aids in keratinocyte migration and proliferation [139]. The hydrogel NU-GELTM is an affordable, transparent, and amorphous gel. Sodium alginate, an ingredient in this hydrogel, efficiently eliminates slough and injured tissue. Additionally, it produces the moisture needed for wound healing. The gel’s alginate content raises its absorptive capacity [140]. Dr. Derm Professional Collagen Hydrogel is a hydrogel used as a cosmetic product that aids in skin regeneration and the restoration of the skin’s suppleness, elasticity, and moisture content. Collagen and hyaluronic acid make up this hydrogel. By providing the skin with moisture, hyaluronic acid contributes significantly to skin hydration [141]. In order to postpone photoaging, [142] created a liposome in a hydrogel complex system that improved medication penetration through the skin. Thanks to hydrogel technology, which facilitates skin hydration and helps to relax the skin barrier, medication penetration through the skin has been enhanced [143]. Bausch & Lomb (Vaughan, Canada) produces soft contact lenses for both short- and long-sightedness, including SofLens Daily disposable, Ultra Contact lens, PureVision2 HD, iconnect, and New Biotrue^®^ lenses. Additionally, they manufacture contact lenses for astigmatism, such as SofLens Daily Disposable Toric, SofLens Toric, and PureVision 2, and for presbyopia, including SofLens Multifocal, Bio real ONEday lenses, and Pure Vision 2 [144].

Inspired by pufferfish, MIT researchers created an ingestible hydrogel device [145]. This fast-acting and highly soluble hydrogel is created by encapsulating polyacrylic acid within a hydrogel membrane composed of polyvinyl alcohol. It can be applied for both prolonged drug release and the measurement of stomach temperature. Other uses for this ingestible hydrogel include monitoring drug habits, measuring bio signals, and observing the gastrointestinal tract with the use of a tiny camera linked to the device. Prodigiosin, a cancer medication, was released from a thermosensitive P(NIPA)-based hydrogel for the treatment of breast cancer, according to [146]. Drug release is greatly influenced by the hydrogel’s porosity and crosslinking ratio. Studies of this nature create a significant impact on drug delivery studies utilizing hydrogels. The fabrication technique used in tissue engineering is crucial, and more studies these days are centered on in situ crosslinked hydrogels. By concurrently injecting a hydrogel and cells, ref. [147] developed a bio-pen that can be used for the in-situ creation of hydrogel scaffolds. Hydrogels have been the subject of in-depth research for tissue engineering applications, although the products’ commercialization is still in its early stages. Hydrogels can be chemically modified to incorporate specific recognition elements such as antibodies, enzymes, or aptamers [143] that bind selectively to biomarkers such as hepcidin, interleukins, and C-reactive protein or pancreatic enzymes like amylase and lipase [145]. Upon the binding of the target biomarker with the recognition element, a change occurs in the hydrogel’s properties, such as its swelling behavior or optical characteristics. This change is transduced into a detectable signal such as a color change, fluorescence, or an electrochemical response, which can be measured quantitatively [147]. The resulting signal is then read using a portable device, providing immediate feedback on the presence and concentration of the biomarker. Hydrogel-based biosensors often require only small volumes of biological samples such as blood or urine. This is advantageous in scenarios where sample collection is challenging or when minimizing patient discomfort is a priority.

### Acute Pancreatitis

The timely and accurate diagnosis of pancreatitis is crucial to initiate appropriate treatment and mitigate complications. Traditional diagnostic methods, which include imaging techniques and biochemical assays, can be time-consuming and require sophisticated laboratory infrastructure to make sure reliable results are being derived [148]. In the context of diagnosing a severe disease like pancreatitis, hydrogel-based biosensors can be engineered to detect specific biomarkers associated with the disease as mentioned earlier in this review, enabling accurate POC testing [149].

Various biomarkers present in serum and ISF (interstitial fluid) can be evaluated for the detection of AP. However, not all biomarkers present in serum are evidently quantified for ISF; therefore, two different strategies based on hydrogels can be employed. Numerous biomarkers have been reported that are common to both plasma and ISF. Owing to the fact that ISF does not clot, it can be employed for continuous monitoring, thereby opening options for minimally invasive monitoring methods using microneedles [150].

**Figure 6:**
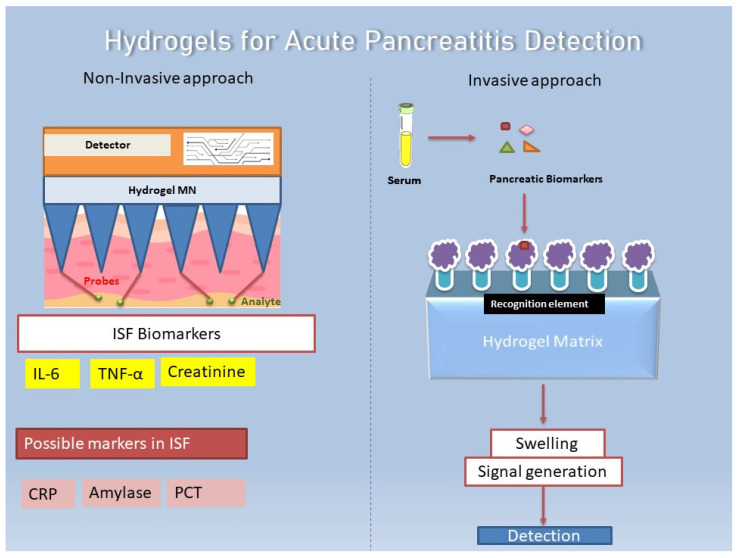
Hydrogel-based biosensors for acute pancreatitis detection. Hypothetical application scenarios providing insights for invasive as well as non-invasive hydrogel-based strategies for the detection of various markers either present in ISF or serum for the assessment and early detection of pancreatitis (Hydrogel MN = microneedle).

Microneedles (MNs) being 15–20 μm in size, are able to traverse the epidermis and reach the ISF, where they can be used for transdermal sampling to detect various biomarkers; when they are integrated with sensory platforms, they can provide a signal readout upon analyte detection [151]. Therefore, non-invasive approaches can be utilized, as illustrated in Figure 6, similar to a report published by [152], where novel MNs along with photonic crystal barcodes were used to perform multiplex detection of three inflammatory cytokines, namely, TNF-α, IL-1β, and IL-6, in ISF, among which IL-6 and TNF-α are known biomarkers for AP [153]. Similarly, in the case of acute kidney Injury (AKI), a complication seen in almost 70% of severe AP patients, it is monitored by increased serum creatinine levels [154], although reports have suggested that creatinine can also be detected in ISF [155]. This opens the possibility that these biomarkers can be detected in ISF for AP.

An invasive approach would rely on the fact that serum drawn from AP patients can be used to detect serum-based biomarkers of AP, with the hydrogels serving as external biosensors conjugated with various biorecognition elements such as enzymes, aptamers, nucleic acids, and ligands [156]. Depending upon the nature of analyte interaction, the hydrogel-based biosensor coupled with a chemical/physical detection system can be used to study and detect these interactions, and even optical and colorimetry-based detection systems can be developed [157].

## 6. Difficulties and Future Perspectives

Hydrogel biosensors represent a very exciting area of research and technology with a lot of potential, but there are several challenges that pose a barrier to their working capacity. Their stability and performance can be affected by environmental conditions like temperature, pH, and ionic strength, which can reduce their sensitivity. Building a biosensor that is strictly specific to a particular biomarker is a task in itself; therefore, hydrogels need to be tailored to interact specifically with the target analytes and not give false results. When producing hydrogel-based biosensors for biomedical applications, one must make sure that they are biocompatible and non-toxic. Ensuring that the materials used are safe for long-term use in biological systems is crucial. Hydrogels can degrade over time, which can affect the lifespan and reliability of the biosensor, so it is essential that hydrogels with improved durability are developed. Keeping these particular facts in mind, future research may focus on developing new hydrogel materials with enhanced properties such as better mechanical strength, chemical stability to increase shelf life, and improved biocompatibility. The development of smart hydrogels that can respond dynamically to environmental changes is also a promising area in the future perspective of this research. Advances in the fields of nanotechnology and micro-fabrication could lead to more precise and scalable production of hydrogel-based biosensors. Techniques like 3D printing may also enable more complex sensor designs. To increase the sensitivity and functionalization of hydrogel biosensors, the incorporation of new types of recognition elements or single transducers apart from fluorescent tags could be an essential step. Apart from the initial idea of a point-of-care kit for hydrogel-based biosensors, the development of flexible and wearable biosensors using hydrogels could lead to new applications in the detection of diseases as severe as pancreatitis.

## 7. Conclusions

Hydrogel-based biosensors offer a promising frontier for the detection and management of pancreatitis due to their unique properties, including high water content, biocompatibility, and adaptability, which make them well suited for developing sensitive and reliable diagnostic tools. The ability to integrate functional elements that can act as substrates for the tagging of biomarkers associated with pancreatitis can potentially lead to the early diagnosis and effective monitoring of the disease. Despite the exciting potential, there are still challenges to address such as improving the stability and specificity of these biosensors and ensuring their practical application in clinical settings without the possibility of an anomaly in the results produced to maximize the efficiency of the biosensor. Continued research and development on this aspect will likely increase the performance of hydrogel-based biosensors, making them an asset in the detection and treatment of pancreatitis. As we look forward, the integration of these biosensors into routine clinical practice could revolutionize how we diagnose and manage this debilitating condition, ultimately leading to better patient outcomes and advancements in personalized medicine. Hydrogel-based biosensors are a novel and promising method for pancreatitis diagnosis and treatment. These biosensors produce highly sensitive and targeted diagnostic tools by combining cutting-edge sensing technology with the biocompatibility, adjustable characteristics, and flexibility of hydrogels. Their reactivity to biological stimuli, including enzymes or pancreatitis-related indicators, improve real-time monitoring and early identification. Furthermore, new potential for point-of-care diagnostics is offered by the integration of hydrogel biosensors with wearable and portable devices, which can improve patient outcomes by implementing prompt interventions. These devices can be further optimized by future developments in material design and sensor integration, which would make them indispensable for the clinical diagnosis and treatment of pancreatitis.

## Figures and Tables

**Table 1:** The causes of acute pancreatitis can be summarized into the following categories.

Mechanical Causes	Toxins and Metabolites	Others
Gallstones	Alcohol	Ischemia
Pancreatic duct obstruction	Hyperlipidemia	Iatrogenic injury
Dysfunction of the sphincter of Oddi	Medications	Infections in the gland
Trauma	Scorpion venom	Hereditary conditions
Ampullary obstruction	Organophosphate poisoning	Autoimmune conditions
Congenital malformations (annular pancreas)	Hypercalcemia	Cystic fibrosis

**Table 2:** Clinically relevant chemical biomarkers to predict acute pancreatitis.

Sl. No.	Biomarker	Cut-Off	Time	References
1.	IL-6	50 pg/mL	24 h	[54]
2.	IL-8	196 pg/mL	Pre-operative	[79]
3.	CRP	150 mg/mL	24 h	[80,81]
4.	Hepcidin	234.4 ng/mL	24 h	[82,83]
5.	Red blood cell distribution width	13.35%	24 h	[84,85]
6.	Blood urea nitrogen	5.945 mg/dL	24 h	[86]

**Table 3:** Summary of the specific types of hydrogels that can be used for the detection of small molecules.

Sr.no	Biomolecule	Hydrogel	Transduction Strategy	Reference
1.	Glucose	Polyaniline	Electrochemical	[118]
		PEG	Electrochemical	[119]
		PVA-vinyl pyridine	Electrochemical	[120]
		Chitosan	Electrochemical	[121]
		Polypyrrole	Electrochemical	[122]
		Alginate	Optical	[123]
2.	Lactate	Polycarbamoyl sulfonate	Electrochemical	[124]
		Albumin–mucin	Electrochemical	[125]
		Chitosan	Electrochemical	[126]
3.	Urea	Polyaniline	Electrochemical	[127]
		Aniline-*co*-o-phenylenediamine	Electrochemical	[128]
4.	Cholesterol	Poly(thionine)	Electrochemical	[129]
		Polypyrrole	Electrochemical	[130]
		Agarose	Electrochemical	[131]
		Polymethacrylate	Optical	[132]
5.	Amylase	Amylopectin and diisocyanate	Quartz crystal microbalance	[133]
6.	Lipase	Pectin hydrogel membrane	Optical	[134]
7.	IL-6	Polypyrrole	Electrochemical	[135]
8.	Creatinine	Photonic crystal hydrogel	Optical	[136]
9.	Trypsin	Gelatin hydrogel	pH based	[137]
10.	C-Reactive protein	Acrylamide, N, N′-methylenebis (acrylamide), 2-methacryloyloxyethyl phosphorylcholine, potassium persulfate	Diffraction-based biogratings	[138]

## References

[1] Pandol S.J., Saluja A.K., Imrie C.W., Banks P.A. (2007). Acute Pancreatitis: Bench to the Bedside. Gastroenterology.

[2] Song Y., Lee S.-H. (2024). Recent Treatment Strategies for Acute Pancreatitis. J. Clin. Med..

[3] Yadav D., Lowenfels A.B. (2013). The Epidemiology of Pancreatitis and Pancreatic Cancer. Gastroenterology.

[4] Coté G.A., Yadav D., Slivka A., Hawes R.H., Anderson M.A., Burton F.R., Brand R.E., Banks P.A., Lewis M.D., Disario J.A. (2011). Alcohol and Smoking as Risk Factors in an Epidemiology Study of Patients With Chronic Pancreatitis. Clin. Gastroenterol. Hepatol..

[5] Cosen-Binker L.I., Lam P.P.L., Binker M.G., Reeve J., Pandol S., Gaisano H.Y. (2007). Alcohol/Cholecystokinin-evoked Pancreatic Acinar Basolateral Exocytosis Is Mediated by Protein Kinase Cα Phosphorylation of Munc18c. J. Biol. Chem..

[6] Göltl P., Murillo K., Simsek O., Wekerle M., Ebert M.P., Schneider A., Hirth M. (2024). Impact of alcohol and smoking cessation on the course of chronic pancreatitis. Alcohol.

[7] Kiss L., Fűr G., Pisipati S., Rajalingamgari P., Ewald N., Singh V., Rakonczay Z. (2023). Mechanisms linking hypertriglyceridemia to acute pancreatitis. Acta Physiol..

[8] Adiamah A., Psaltis E., Crook M., Lobo D.N. (2018). A systematic review of the epidemiology, pathophysiology and current management of hyperlipidaemic pancreatitis. Clin. Nutr..

[9] Napier S., Thomas M. (2006). 36 year old man presenting with pancreatitis and a history of recent commencement of orlistat case report. Nutr. J..

[10] Jones M.R., Hall O.M., Kaye A.M., Kaye A.D. (2015). Drug-induced acute pancreatitis: A review. Ochsner J..

[11] Suzuki M. (2014). Acute pancreatitis in children and adolescents. World J. Gastrointest. Pathophysiol..

[12] Walkowska J., Zielinska N., Tubbs R.S., Podgórski M., Dłubek-Ruxer J., Olewnik Ł. (2022). Diagnosis and treatment of acute pancreatitis. Diagnostics.

[13] Beyer G., Hoffmeister A., Lorenz P., Lynen P., Lerch M.M., Mayerle J. (2022). Clinical practice guideline: Acute and chronic pancreatitis. Dtsch. Aerzteblatt Online.

[14] Daurai B., Baruah A.J., Gogoi M. (2024). Enzymatic Biosensors for Detection of Pancreatitis. Enzyme-Based Biosensors: Recent Advances and Applications in Healthcare.

[15] Jia W., Xu L., Xu W., Yang M., Zhang Y. (2022). Application of nanotechnology in the diagnosis and treatment of acute pancreatitis. Nanoscale Adv..

[16] Sun S., Chen J. (2024). Recent Advances in Hydrogel-Based Biosensors for Cancer Detection. ACS Appl. Mater. Interfaces.

[17] Völlmecke K., Afroz R., Bierbach S., Brenker L.J., Frücht S., Glass A., Giebelhaus R., Hoppe A., Kanemaru K., Lazarek M. (2022). Hydrogel-Based Biosensors. Gels.

[18] Liu R., Nie Q., Wang Y., Wu Y., Tu Y., Xie C., Xiao X., You R., Lu Y. (2024). Diaper-based wearable SERS sensing system with a silver nano dual-structure composite hydrogel for the detection of biomarkers and pH in urine. Chem. Eng. J..

[19] Liu Y., Huang T., Qian Z., Chen W. (2023). Extensible and swellable hydrogel-forming microneedles for deep point-of-care sampling and drug deployment. Chin. Chem. Lett..

[20] Mederos M.A., Reber H.A., Girgis M.D. (2021). Acute Pancreatitis. JAMA.

[21] Lankisch P.G., Apte M., Banks P.A. (2015). Acute pancreatitis. Lancet.

[22] Yang A.L., McNabb-Baltar J. (2020). Hypertriglyceridemia and acute pancreatitis. Pancreatology.

[23] Szatmary P., Grammatikopoulos T., Cai W., Huang W., Mukherjee R., Halloran C., Beyer G., Sutton R. (2022). Acute Pancreatitis: Diagnosis and Treatment. Drugs.

[24] Khatua B., El-Kurdi B., Singh V.P. (2017). Obesity and pancreatitis. Curr. Opin. Gastroenterol..

[25] Richardson A., Park W.G. (2021). Acute pancreatitis and diabetes mellitus: A review. Korean J. Intern. Med..

[26] Boxhoorn L., Voermans R.P., Bouwense S.A., Bruno M.J., Verdonk R.C., Boermeester M.A., van Santvoort H.C., Besselink M.G. (2020). Acute pancreatitis. Lancet.

[27] Lee P.J., Papachristou G.I. (2019). New insights into acute pancreatitis. Nat. Rev. Gastroenterol. Hepatol..

[28] Habtezion A., Gukovskaya A.S., Pandol S.J. (2019). Acute Pancreatitis: A Multifaceted Set of Organelle and Cellular Interactions. Gastroenterology.

[29] Maatman T.K., Zyromski N.J. (2020). Chronic Pancreatitis. Curr. Probl. Surg..

[30] Zierke L., John D., Gischke M., Tran Q.T., Sendler M., Weiss F.U., Bornscheuer U.T., Ritter C., Lerch M.M., Aghdassi A.A. (2024). Initiation of acute pancreatitis in mice is independent of fusion between lysosomes and zymogen granules. Cell. Mol. Life Sci..

[31] Krüger B., Albrecht E., Lerch M.M. (2000). The Role of Intracellular Calcium Signaling in Premature Protease Activation and the Onset of Pancreatitis. Am. J. Pathol..

[32] Antonucci L., Fagman J.B., Kim J.Y., Todoric J., Gukovsky I., Mackey M., Ellisman M.H., Karin M. (2015). Basal autophagy maintains pancreatic acinar cell homeostasis and protein synthesis and prevents ER stress. Proc. Natl. Acad. Sci. USA.

[33] Diakopoulos K.N., Lesina M., Wörmann S., Song L., Aichler M., Schild L., Artati A., Römisch-Margl W., Wartmann T., Fischer R. (2015). Impaired Autophagy Induces Chronic Atrophic Pancreatitis in Mice via Sex- and Nutrition-Dependent Processes. Gastroenterology.

[34] Hennig P., Fenini G., Di Filippo M., Karakaya T., Beer H.-D. (2021). The Pathways Underlying the Multiple Roles of p62 in Inflammation and Cancer. Biomedicines.

[35] Baer J.M., Zuo C., Kang L.-I., de la Lastra A.A., Borcherding N.C., Knolhoff B.L., Bogner S.J., Zhu Y., Yang L., Laurent J. (2023). Fibrosis induced by resident macrophages has divergent roles in pancreas inflammatory injury and PDAC. Nat. Immunol..

[36] Wang K., Zhao A., Tayier D., Tan K., Song W., Cheng Q., Li X., Chen Z., Wei Q., Yuan Y. (2023). Activation of AMPK ameliorates acute severe pancreatitis by suppressing pancreatic acinar cell necroptosis in obese mice models. Cell Death Discov..

[37] Saluja A., Dudeja V., Dawra R., Sah R.P. (2019). Early Intra-Acinar Events in Pathogenesis of Pancreatitis. Gastroenterology.

[38] Watanabe T., Kudo M., Strober W. (2017). Immunopathogenesis of pancreatitis. Mucosal Immunol..

[39] Zhou Y., Huang X., Jin Y., Qiu M., Ambe P.C., Basharat Z., Hong W. (2024). The role of mitochondrial damage-associated molecular patterns in acute pancreatitis. Biomed. Pharmacother..

[40] Yasuda T., Ueda T., Shinzeki M., Sawa H., Nakajima T., Takeyama Y., Kuroda Y. (2007). Increase of High-Mobility Group Box Chromosomal Protein 1 in Blood and Injured Organs in Experimental Severe Acute Pancreatitis. Pancreas.

[41] Hoque R., Malik A.F., Gorelick F., Mehal W.Z. (2012). Sterile Inflammatory Response in Acute Pancreatitis. Pancreas.

[42] Kang R., Chen R., Xie M., Cao L., Lotze M.T., Tang D., Zeh H.J. (2016). The Receptor for Advanced Glycation End Products Activates the AIM2 Inflammasome in Acute Pancreatitis. J. Immunol..

[43] Strum W.B., Boland C.R. (2023). Advances in acute and chronic pancreatitis. World J. Gastroenterol..

[44] van Geenen E.J.M., van der Peet D.L., Bhagirath P., Mulder C.J.J., Bruno M.J. (2010). Etiology and diagnosis of acute biliary pancreatitis. Nat. Rev. Gastroenterol. Hepatol..

[45] Wang G.-J., Li Y., Zhou Z.-G., Wang C., Meng W.-J. (2010). Integrity of the pancreatic duct-acinar system in the pathogenesis of acute pancreatitis. Hepatobiliary Pancreat. Dis. Int..

[46] Biczo G., Vegh E.T., Shalbueva N., Mareninova O.A., Elperin J., Lotshaw E., Gretler S., Lugea A., Malla S.R., Dawson D. (2018). Mitochondrial Dysfunction, Through Impaired Autophagy, Leads to Endoplasmic Reticulum Stress, Deregulated Lipid Metabolism, and Pancreatitis in Animal Models. Gastroenterology.

[47] Wang G.-J., Gao C.-F., Wei D., Wang C., Ding S.-Q. (2009). Acute pancreatitis: Etiology and common pathogenesis. World J. Gastroenterol..

[48] Sternby H., Hartman H., Thorlacius H., Regnér S. (2021). The Initial Course of IL1β, IL-6, IL-8, IL-10, IL-12, IFN-γ and TNF-α with Regard to Severity Grade in Acute Pancreatitis. Biomolecules.

[49] Yang H., Cao R., Zhou F., Wang B., Xu Q., Li R., Zhang C., Xu H. (2024). The role of Interleukin-22 in severe acute pancreatitis. Mol. Med..

[50] Zhang C., Shi D., Li X., Yuan J. (2022). Microfluidic electrochemical magneto immunosensor for ultrasensitive detection of interleukin-6 based on hybrid of AuNPs and graphene. Talanta.

[51] Wang W., Han R., Chen M., Luo X. (2021). Antifouling Peptide Hydrogel Based Electrochemical Biosensors for Highly Sensitive Detection of Cancer Biomarker HER2 in Human Serum. Anal. Chem..

[52] Paajanens H., Laato M., Jaakkola M., Pulkki K., Niinikoski J., Nordback I. (1995). Serum tumour necrosis factor compared with C-reactive protein in the early assessment of severity of acute pancreatitis. Br. J. Surg..

[53] Sánchez-Tirado E., Salvo C., González-Cortés A., Yáñez-Sedeño P., Langa F., Pingarrón J. (2017). Electrochemical immunosensor for simultaneous determination of interleukin-1 beta and tumor necrosis factor alpha in serum and saliva using dual screen printed electrodes modified with functionalized double–walled carbon nanotubes. Anal. Chim. Acta.

[54] Khanna A.K., Meher S., Prakash S., Tiwary S.K., Singh U., Srivastava A., Dixit V.K. (2013). Comparison of Ranson, Glasgow, MOSS, SIRS, BISAP, APACHE-II, CTSI Scores, IL-6, CRP, and Procalcitonin in Predicting Severity, Organ Failure, Pancreatic Necrosis, and Mortality in Acute Pancreatitis. HPB Surg..

[55] Piñerúa-Gonsálvez J.F., Ruiz Rebollo M.L., Zambrano-Infantino R.D.C., Rizzo-Rodríguez M.A., Fernández-Salazar L. (2023). Value of CRP/albumin ratio as a prognostic marker of acute pancreatitis: A retrospective study. Rev. Esp. Enfermedades Dig..

[56] Bryan T., Luo X., Bueno P.R., Davis J.J. (2013). An optimised electrochemical biosensor for the label-free detection of C-reactive protein in blood. Biosens. Bioelectron..

[57] Guillem P., Bustos R.-H., Garzon V., Munoz A., Juez G. (2021). A low-cost electrochemical biosensor platform for C-reactive protein detection. Sens. Bio-Sens. Res..

[58] Woo S.M., Noh M.H., Kim B.G., Ter Hsing C., Han J.S., Ryu S.H., Seo J.M., Yoon H.A., Jang J.S., Choi S.R. (2011). Comparison of Serum Procalcitonin with Ranson, APACHE-II, Glasgow and Balthazar CT Severity Index Scores in Predicting Severity of Acute Pancreatitis. Korean J. Gastroenterol..

[59] Párniczky A., Lantos T., Tóth E.M., Szakács Z., Gódi S., Hágendorn R., Illés D., Koncz B., Márta K., Mikó A. (2019). Antibiotic therapy in acute pancreatitis: From global overuse to evidence based recommendations. Pancreatology.

[60] Siriwardena A.K., Jegatheeswaran S., Mason J.M., Baltatzis M., Chan A., Sheen A.J., O’reilly D., Jamdar S., Deshpande R., Carino N.d.L. (2019). PROCalcitonin-based algorithm for antibiotic use in Acute Pancreatitis (PROCAP): Study protocol for a randomised controlled trial. Trials.

[61] Mofidi R., Suttie S.A., Patil P.V., Ogston S., Parks R.W. (2009). The value of procalcitonin at predicting the severity of acute pancreatitis and development of infected pancreatic necrosis: Systematic review. Surgery.

[62] Ida S., Fujimura Y., Hirota M., Imamura Y., Ozaki N., Suyama K., Hashimoto D., Ohmuraya M., Tanaka H., Takamori H. (2009). Significance of endothelial molecular markers in the evaluation of the severity of acute pancreatitis. Surg. Today.

[63] Luo P., Xie Y., He X., Zhang W., Tan L. (2024). Microvolumetric determination of thrombomodulin based on competitive immunoreaction using a portable glucometer. Microchim. Acta.

[64] Gan S.I., Romagnuolo J. (2004). Admission Hematocrit: A Simple, Useful and Early Predictor of Severe Pancreatitis. Dig. Dis. Sci..

[65] Parsa N., Faghih M., Gonzalez F.G., Moran R.A., Kamal A., Jalaly N.Y., Al-Grain H., Akshintala V.S., Makary M.A., Khashab M.A. (2019). Early Hemoconcentration Is Associated With Increased Opioid Use in Hospitalized Patients with Acute Pancreatitis. Pancreas.

[66] Schult L., Halbgebauer R., Karasu E., Huber-Lang M. (2023). Glomerular injury after trauma, burn, and sepsis. J. Nephrol..

[67] Garg P.K., Singh V.P. (2019). Organ Failure Due to Systemic Injury in Acute Pancreatitis. Gastroenterology.

[68] Oz H.S. (2012). Gene expression profiling and endothelin in acute experimental pancreatitis. World J. Gastroenterol..

[69] Xu X., Ai F., Huang M. (2020). Deceased serum bilirubin and albumin levels in the assessment of severity and mortality in patients with acute pancreatitis. Int. J. Med. Sci..

[70] Liu J., Wang G., Liu Y., Huang L., Xu X., Wang J. (2019). Effects of Somatostatin Combined with Pantoprazole on Serum C-Reactive Protein and Intercellular Adhesion Molecule-1 in Severe Acute Pancreatitis. J. Coll. Physicians Surg. Pak..

[71] Ou X., Cheng Z., Liu T.B., Tang Z., Huang W., Szatmary P.M., Zheng S., Sutton R.D., Toh C.H., Zhang N. (2015). Circulating Histone Levels Reflect Disease Severity in Animal Models of Acute Pancreatitis. Pancreas.

[72] Orfanos S.E., Kotanidou A., Glynos C., Athanasiou C., Tsigkos S., Dimopoulou I., Sotiropoulou C., Zakynthinos S., Armaganidis A., Papapetropoulos A. (2007). Angiopoietin-2 is increased in severe sepsis: Correlation with inflammatory mediators. Crit. Care Med..

[73] Shyam R., Sachan R., Patel M., Bharti H., Gupta K., Parihar A. (2020). Evaluation of Serum Cystatin C as an Early Biomarker of Acute Kidney Injury in Patients with Acute Pancreatitis. Indian J. Crit. Care Med..

[74] Ugurlu E.T., Tercan M. (2022). The role of biomarkers in the early diagnosis of acute kidney injury associated with acute pancreatitis: Evidence from 582 cases. Turk. J. Trauma Emerg. Surg..

[75] de Pretis N., Amodio A., De Marchi G., Marconato E., Ciccocioppo R., Frulloni L. (2022). The role of serological biomarkers in the diagnosis and management of autoimmune pancreatitis. Expert Rev. Clin. Immunol..

[76] Lin Z., Liu M., Xing W., Wang F., Zhang H., Wei X., Schmitthenner H., Xie X., Xia X., Yang J. (2023). A near-infrared fluorescence-enhancing plasmonic biosensing microarray identifies soluble PD-L1 and ICAM-1 as predictive checkpoint biomarkers for cancer immunotherapy. Biosens. Bioelectron..

[77] Zielinska Z., Oldak L., Guszcz T., Hermanowicz A., Gorodkiewicz E. (2024). SPRi Biosensor for Simultaneous Determi-nation of HIF-1α, Angiopoietin-2, and Interleukin-1β in Blood Plasma. Sensors.

[78] Tzianni Ε.I., Moutsios I., Moschovas D., Avgeropoulos A., Govaris K., Panagiotidis L., Prodromidis M.I. (2022). Smartphone paired SIM card-type integrated creatinine biosensor. Biosens. Bioelectron..

[79] Khamdamov B.Z., Ganiev A.A., Khamdamov I.B. (2023). The role of cytokines in the immunopatogenesis of acute pancreatitis. J. Surv. Fish. Sci..

[80] Mumin A., Kabir A.S., Noor R.A., Rahman U., Al Amin A. (2024). Role of C-Reactive Protein (CRP) and Neutrophil Lymphocyte Ratio (NLR) in detecting severity & Predicting outcome of Acute Pancreatitis patients. Dinkum J. Med. Innov..

[81] Cardoso F.S., Ricardo L.B., Oliveira A.M., Canena J.M., Horta D.V., Papoila A.L., Deus J.R. (2013). C-reactive protein prognostic accuracy in acute pancreatitis: Timing of measurement and cutoff points. Eur. J. Gastroenterol. Hepatol..

[82] Arabul M., Celik M., Aslan O., Torun S., Beyazit Y., Alper E., Kandemir A., Ünsal B. (2013). Hepcidin as a predictor of disease severity in acute pancreatitis: A single center prospective study. Hepatogastroenterology.

[83] Kimita W.M., Ko J.M., Li X.M., Bharmal S.H.M., Petrov M.S. (2022). Associations Between Iron Homeostasis and Pancreatic Enzymes After an Attack of Pancreatitis. Pancreas.

[84] Yao J., Lv G. (2014). Association between red cell distribution width and acute pancreatitis: A cross-sectional study. BMJ Open.

[85] Han T., Cheng T., Liao Y., He Y., Liu B., Lai Q., Pan P., Liu J., Cao Y., Yu H. (2022). The ratio of red blood cell distribution width to serum calcium predicts severity of patients with acute pancreatitis. Am. J. Emerg. Med..

[86] Lin S., Hong W., Basharat Z., Wang Q., Pan J., Zhou M. (2017). Blood urea nitrogen as a predictor of severe acute pancreatitis based on the revised Atlanta criteria: Timing of measurement and cutoff points. Can. J. Gastroenterol. Hepatol..

[87] Hwang H.J., Ryu M.Y., Park C.Y., Ahn J., Park H.G., Choi C., Ha S.-D., Park T.J., Park J.P. (2017). High sensitive and selective electrochemical biosensor: Label-free detection of human norovirus using affinity peptide as molecular binder. Biosens. Bioelectron..

[88] Cao H., Duan L., Zhang Y., Cao J., Zhang K. (2021). Current hydrogel advances in physicochemical and biological response-driven biomedical application diversity. Signal Transduct. Target. Ther..

[89] Ahmed B., Reiche C.F., Magda J.J., Solzbacher F., Körner J. (2024). Smart Hydrogel Swelling State Detection Based on a Power-Transfer Transduction Principle. ACS Appl. Polym. Mater..

[90] Naresh V., Lee N. (2021). A Review on Biosensors and Recent Development of Nanostructured Materials-Enabled Biosensors. Sensors.

[91] Zou R., Li H., Shi J., Sun C., Lu G., Yan X. (2024). Dual-enhanced enzyme cascade hybrid hydrogel for the construction of optical biosensor. Biosens. Bioelectron..

[92] Govindaraj M., Srivastava A., Muthukumaran M.K., Tsai P.-C., Lin Y.-C., Raja B.K., Rajendran J., Ponnusamy V.K., Selvi J.A. (2023). Current advancements and prospects of enzymatic and non-enzymatic electrochemical glucose sensors. Int. J. Biol. Macromol..

[93] Wei Y., Zeng Q., Wang M., Huang J., Guo X., Wang L. (2019). Near-Infrared light-responsive electrochemical protein imprinting biosensor based on a shape memory conducting hydrogel. Biosens. Bioelectron..

[94] Ryu J.J., Jang C.H. (2023). A liquid crystal-based biosensor for sensitive detection of tumor necrosis factor-alpha. Mikrochim Acta..

[95] Kizhakkeveettil A., Parla J., Patwardhan K., Sharma A., Sharma S. (2024). History, Present and Prospect of Ayurveda. History, Present and Prospect of World Traditional Medicine.

[96] Chattopadhyay K., Wang H., Kaur J., Nalbant G., Almaqhawi A., Kundakci B., Panniyammakal J., Heinrich M., Lewis S.A., Greenfield S.M. (2022). Effectiveness and Safety of Ayurvedic Medicines in Type 2 Diabetes Mellitus Management: A Systematic Review and Meta-Analysis. Front. Pharmacol..

[97] Bhattacharya S., Anil Bansal S., Khanna V., Balakrishnan N., Gupta P. (2024). Application of a Novel Nanotherapeutic Strategy in Ayurvedic Treatment. Emerging Applications of Novel Nanoparticles. Lecture Notes in Nanoscale Science and Technology.

[98] Prakash B., Prakash S., Sati S.T., Negi N. (2024). Ayurvedic treatment protocol in the management of pancreatitis: A nonrandomized observational study. Int. J. Ayurveda Res..

[99] Sokiya G., Painuly P., Negi M., Sharma G. (2024). An Ayurvedic approach towards management of Acute Pancreatitis: A Case Study. J. Ayurveda Integr. Med. Sci..

[100] Zhang C.H., Jia C.Y., Li W., Zhao F.P., Yu Q.N., Yu J.Y., Wang Y.H., Yang R.P. (2015). Preparation of Sanqi Hydrogel Patch used for setting a bone and study on its transdermal permeability in vitro. Zhongcaoyao.

[101] Herrmann A., Haag R., Schedler U. (2021). Hydrogels and Their Role in Biosensing Applications. Adv. Healthc. Mater..

[102] Tavakoli J., Tang Y. (2017). Hydrogel Based Sensors for Biomedical Applications: An Updated Review. Polymers.

[103] Danks A.E., Hall S.R., Schnepp Z.J.M.H. (2016). The evolution of ‘sol–gel’chemistry as a technique for materials synthesis. Mater. Horiz..

[104] Echalier C., Jebors S., Laconde G., Brunel L., Verdié P., Causse L., Bethry A., Legrand B., Van Den Berghe H., Garric X. (2017). Sol–gel synthesis of collagen-inspired peptide hydrogel. Mater. Today.

[105] Zhong Y., Lin Q., Yu H., Shao L., Cui X., Pang Q., Zhu Y., Hou R. (2024). Construction methods and biomedical applications of PVA-based hydrogels. Front. Chem..

[106] Tsai H.-C., Doong R.-A. (2007). Preparation and characterization of urease-encapsulated biosensors in poly(vinyl alcohol)-modified silica sol–gel materials. Biosens. Bioelectron..

[107] Muñoz E.M., Yu H., Hallock J., Edens R.E., Linhardt R.J. (2005). Poly(ethylene glycol)-based biosensor chip to study heparin–protein interactions. Anal. Biochem..

[108] Riedel T., Riedelová-Reicheltová Z., Májek P., Rodriguez-Emmenegger C., Houska M., Dyr J.E., Brynda E. (2013). Complete Identification of Proteins Responsible for Human Blood Plasma Fouling on Poly(ethylene glycol)-Based Surfaces. Langmuir.

[109] Lowe S., O’Brien-Simpson N.M., Connal L.A. (2014). Antibiofouling polymer interfaces: Poly(ethylene glycol) and other promising candidates. Polym. Chem..

[110] Yin M., Yao M., Gao S., Zhang A.P., Tam H., Wai P.A. (2015). Rapid 3D Patterning of Poly(acrylic acid) Ionic Hydrogel for Miniature pH Sensors. Adv. Mater..

[111] Majumdar S., Dey J., Adhikari B. (2006). Taste sensing with polyacrylic acid grafted cellulose membrane. Talanta.

[112] Zhao Y., Sun S. (2024). Adhesive and conductive hydrogels based on poly(acrylic acid) composites for application as flexible biosensors. Colloids Surf. A Physicochem. Eng. Asp..

[113] Wang X., Han M., Bao J., Tu W., Dai Z. (2012). A superoxide anion biosensor based on direct electron transfer of superoxide dismutase on sodium alginate sol–gel film and its application to monitoring of living cells. Anal. Chim. Acta.

[114] Polyak B., Geresh S., Marks R.S. (2004). Synthesis and Characterization of a Biotin-Alginate Conjugate and Its Application in a Biosensor Construction. Biomacromolecules.

[115] Kesharwani P., Alexander A., Shukla R., Jain S., Bisht A., Kumari K., Verma K., Sharma S. (2024). Tissue regeneration properties of hydrogels derived from biological macromolecules: A review. Int. J. Biol. Macromol..

[116] Yang M., Jeong J.-M., Lee K.G., Kim D.H., Lee S.J., Choi B.G. (2017). Hierarchical porous microspheres of the Co(3)O(4)@graphene with enhanced electrocatalytic performance for electrochemical biosensors. Biosens. Bioelectron..

[117] Nguyen H.H., Lee S.H., Lee U.J., Fermin C.D., Kim M. (2019). Immobilized Enzymes in Biosensor Applications. Materials.

[118] Zhai D., Liu B., Shi Y., Pan L., Wang Y., Li W., Zhang R., Yu G. (2013). Highly Sensitive Glucose Sensor Based on Pt Nanoparticle/Polyaniline Hydrogel Heterostructures. ACS Nano.

[119] Mano N., Yoo J.E., Tarver J., Loo Y.-L., Heller A. (2007). An Electron-Conducting Cross-Linked Polyaniline-Based Redox Hydrogel, Formed in One Step at pH 7.2, Wires Glucose Oxidase. J. Am. Chem. Soc..

[120] Russell R.J., Pishko M.V., Gefrides C.C., McShane M.J., Coté G.L. (1999). A Fluorescence-Based Glucose Biosensor Using Concanavalin A and Dextran Encapsulated in a Poly(ethylene glycol) Hydrogel. Anal. Chem..

[121] Wang B., Li B., Deng Q., Dong S. (1998). Amperometric Glucose Biosensor Based on Sol−Gel Organic−Inorganic Hybrid Material. Anal. Chem..

[122] Reiter S., Habermüller K., Schuhmann W. (2001). A reagentless glucose biosensor based on glucose oxidase entrapped into osmium-complex modified polypyrrole films. Sens. Actuators B Chem..

[123] Bornhoeft L.R., Biswas A., McShane M.J. (2017). Composite Hydrogels with Engineered Microdomains for Optical Glucose Sensing at Low Oxygen Conditions. Biosensors.

[124] Kwan R.C., Hon P.Y., Mak K.K., Renneberg R. (2004). Amperometric determination of lactate with novel trienzyme/poly(carbamoyl) sulfonate hydrogel-based sensor. Biosens. Bioelectron..

[125] Romero M.R., Garay F., Baruzzi A.M. (2007). Design and optimization of a lactate amperometric biosensor based on lactate oxidase cross-linked with polymeric matrixes. Sens. Actuators B Chem..

[126] Zanini V.P., de Mishima B.L., Solís V. (2011). An amperometric biosensor based on lactate oxidase immobilized in laponite–chitosan hydrogel on a glassy carbon electrode. Application to the analysis of l-lactate in food samples. Sens. Actuators B Chem..

[127] Zhybak M., Beni V., Vagin M., Dempsey E., Turner A., Korpan Y. (2016). Creatinine and urea biosensors based on a novel ammonium ion-selective copper-polyaniline nano-composite. Biosens. Bioelectron..

[128] Ma W.-J., Luo C.-H., Lin J.-L., Chou S.-H., Chen P.-H., Syu M.-J., Kuo S.-H., Lai S.-C. (2016). A Portable Low-Power Acquisition System with a Urease Bioelectrochemical Sensor for Potentiometric Detection of Urea Concentrations. Sensors.

[129] Rahman M., Li X.-B., Kim J., Lim B.O., Ahammad A.S., Lee J.-J. (2014). A cholesterol biosensor based on a bi-enzyme immobilized on conducting poly(thionine) film. Sens. Actuators B Chem..

[130] Brahim S., Narinesingh D., Guiseppi-Elie A. (2001). Amperometric determination of cholesterol in serum using a biosensor of cholesterol oxidase contained within a polypyrrole–hydrogel membrane. Anal. Chim. Acta.

[131] Shumyantseva V., Deluca G., Bulko T., Carrara S., Nicolini C., Usanov S.A., Archakov A. (2004). Cholesterol amperometric biosensor based on cytochrome P450scc. Biosens. Bioelectron..

[132] Soylemez S., Udum Y.A., Kesik M., Hızlıateş C.G., Ergun Y., Toppare L. (2015). Electrochemical and optical properties of a conducting polymer and its use in a novel biosensor for the detection of cholesterol. Sens. Actuators B Chem..

[133] Gibbs M.J., Biela A., Krause S. (2015). α-Amylase sensor based on the degradation of oligosaccharide hydrogel films monitored with a quartz crystal sensor. Biosens. Bioelectron..

[134] Hasanah U., Sani N.D.M., Heng L.Y., Idroes R., Safitri E. (2019). Construction of a Hydrogel Pectin-Based Triglyceride Optical Biosensor with Immobilized Lipase Enzymes. Biosensors.

[135] Shi D., Zhang C., Li X., Yuan J. (2023). An electrochemical paper-based hydrogel immunosensor to monitor serum cytokine for predicting the severity of COVID-19 patients. Biosens. Bioelectron..

[136] Rafique B., Khan R.U., Rizvi A.S., Irfan M., Murtaza G., Qiu L., Xue M., Meng Z. (2023). Creatinine Imprinted Photonic Crystals Hydrogel Sensor. Arab. J. Chem..

[137] Ping J., Wu W., Qi L., Liu J., Liu J., Zhao B., Wang Q., Yu L., Lin J.-M., Hu Q. (2021). Hydrogel-assisted paper-based lateral flow sensor for the detection of trypsin in human serum. Biosens. Bioelectron..

[138] Lucío M.I., Montoto A.H., Fernández E., Alamri S., Kunze T., Bañuls M.-J., Maquieira Á. (2021). Label-free detection of C-Reactive protein using bioresponsive hydrogel-based surface relief diffraction gratings. Biosens. Bioelectron..

[139] Catanzano O., D’esposito V., Acierno S., Ambrosio M., De Caro C., Avagliano C., Russo P., Russo R., Miro A., Ungaro F. (2015). Alginate–hyaluronan composite hydrogels accelerate wound healing process. Carbohydr. Polym..

[140] Aswathy S., Narendrakumar U., Manjubala I. (2020). Commercial hydrogels for biomedical applications. Heliyon.

[141] Papakonstantinou E., Roth M., Karakiulakis G. (2012). A key molecule in skin aging Hyaluronic acid. Dermatoendocrinology.

[142] Kim S.J., Kwon S.S., Jeon S.H., Yu E.R., Park S.N. (2014). Enhanced skin delivery of liquiritigenin and liquiritin-loaded liposome-in-hydrogel complex system. Int. J. Cosmet. Sci..

[143] Wu Y., Midinov B., White R.J. (2019). Electrochemical Aptamer-Based Sensor for Real-Time Monitoring of Insulin. ACS Sens..

[144] Lichtman J.W., Conchello J.-A. (2005). Fluorescence microscopy. Nat. Methods.

[145] Liu X., Steiger C., Lin S., Parada G.A., Liu J., Chan H.F., Yuk H., Phan N.V., Collins J., Tamang S. (2019). Ingestible hydrogel device. Nat. Commun..

[146] Danyuo Y., Ani C.J., Salifu A.A., Obayemi J.D., Dozie-Nwachukwu S., Obanawu V.O., Akpan U.M., Odusanya O.S., Abade-Abugre M., McBagonluri F. (2019). Anomalous Release Kinetics of Prodigiosin from Poly-N-Isopropyl-Acrylamid based Hydrogels for The Treatment of Triple Negative Breast Cancer. Sci. Rep..

[147] O’Connell C.D., Di Bella C., Thompson F., Augustine C., Beirne S., Cornock R., Richards C.J., Chung J., Gambhir S., Yue Z. (2016). Development of the Biopen: A handheld device for surgical printing of adipose stem cells at a chondral wound site. Biofabrication.

[148] Smotkin J., Tenner S. (2002). Laboratory Diagnostic Tests in Acute Pancreatitis. J. Clin. Gastroenterol..

[149] Simha A., Saroch A., Pannu A.K., Dhibar D.P., Sharma N., Singh H., Sharma V. (2021). Utility of Point-Of-Care Urine Trypsinogen Dipstick Test for Diagnosing Acute Pancreatitis in An Emergency Unit. Biomark. Med..

[150] Samant P.P., Niedzwiecki M.M., Raviele N., Tran V., Mena-Lapaix J., Walker D.I., Felner E.I., Jones D.P., Miller G.W., Prausnitz M.R. (2020). Sampling interstitial fluid from human skin using a microneedle patch. Sci. Transl. Med..

[151] Lu H., Zada S., Yang L., Dong H. (2022). Microneedle-Based Device for Biological Analysis. Front. Bioeng. Biotechnol..

[152] Wang Y., Shang L., Bian F., Zhang X., Wang S., Zhou M., Zhao Y. (2019). Hollow colloid assembled photonic crystal clusters as suspension barcodes for multiplex bioassays. Small.

[153] Meher S., Mishra T.S., Sasmal P.K., Rath S., Sharma R., Rout B., Sahu M.K. (2015). Role of biomarkers in diagnosis and prognostic evaluation of acute pancreatitis. J. Biomark..

[154] Wajda J., Dumnicka P., Maraj M., Ceranowicz P., Kuźniewski M., Kuśnierz-Cabala B. (2019). Potential Prognostic Markers of Acute Kidney Injury in the Early Phase of Acute Pancreatitis. Int. J. Mol. Sci..

[155] Chachaj A., Matkowski R., Gröbner G., Szuba A., Dudka I. (2020). Metabolomics of Interstitial Fluid, Plasma and Urine in Patients with Arterial Hypertension: New Insights into the Underlying Mechanisms. Diagnostics.

[156] Jung I.Y., Kim J.S., Choi B.R., Lee K., Lee H. (2017). Hydrogel Based Biosensors for In Vitro Diagnostics of Biochemicals, Proteins, and Genes. Adv. Healthc. Mater..

[157] Bae J., Park J., Kim S., Cho H., Kim H.J., Park S., Shin D.S. (2020). Tailored hydrogels for biosensor applications. J. Ind. Eng. Chem..

